# Sensitization to Indigenous Pollen and Molds and Other Outdoor and Indoor Allergens in Allergic Patients From Saudi Arabia, United Arab Emirates, and Sudan

**DOI:** 10.1097/WOX.0b013e31825a73cd

**Published:** 2012-06-15

**Authors:** SM Hasnain, AR Al-Frayh, JL Subiza, Enrique Fernández-Caldas, M Casanovas, T Geith, MO Gad-El-Rab, E Koshak, H Al-Mehdar, S Al-Sowaidi, H Al-Matar, R Khouqeer, K Al-Abbad, M Al-Yamani, E Alaqi, OA Musa, S Al-Sedairy

**Affiliations:** 1 King Faisal Specialist Hospital & Research Center, Riyadh, Saudi Arabia; 2 College of Medicine, King Saud University, Riyadh, Saudi Arabia; 3 Inmunotek, Madrid, Spain; 4 Elaj Medical Services, Riyadh and Khamis Mushait, Saudi Arabia; 5 Faculty of Medicine, King Abdulaziz University Hospital, Jeddah, Saudi Arabia; 6 King Fahad Medical Research Centre, King Abdulaziz University, Jeddah, Saudi Arabia; 7 Faculty of Medicine and Health Sciences, UAE University, Abu Dhabi, United Arab Emirates; 8 Al Imam Abdulrahman Bin Faisal Hospital, Dammam, Saudi Arabia; 9 Saad Specialist Hospital, Al-Khobar, Saudi Arabia; 10 Maternity & Children's Hospital, Dammam, Saudi Arabia; 11 Children's Hospital, Riyadh Medical Complex, Riyadh, Saudi Arabia; 12 Faculty of Medicine, The National Ribat University, Khartoum, Sudan

**Keywords:** allergens, diagnostics, bronchial asthma, allergic rhinitis, Saudi Arabia, *Salsola*, *Prosopis*, mites

## Abstract

**Background:**

Airborne allergens vary from one climatic region to another. Therefore, it is important to analyze the environment of the region to select the most prevalent allergens for the diagnosis and treatment of allergic patients.

**Objective:**

To evaluate the prevalence of positive skin tests to pollen and fungal allergens collected from local indigenous plants or isolated molds, as well as other outdoor and indoor allergens in allergic patients in 6 different geographical areas in the Kingdom of Saudi Arabia (KSA), the United Arab Emirates, and Sudan.

**Materials and methods:**

Four hundred ninety-two consecutive patients evaluated at different Allergy Clinics (276 women and 256 men; mean age, 30 years) participated in this study. The selection of indigenous allergens was based on research findings in different areas from Riyadh and adjoining areas. Indigenous raw material for pollen grains was collected from the desert near the capital city of Riyadh, KSA. The following plants were included: *Chenopodium murale, Salsola imbricata, Rumex vesicarius, Ricinus communis, Artiplex nummularia, Amaranthus viridis, Artemisia monosperma, Plantago boissieri*, and *Prosopis juliflora*. Indigenous molds were isolated from air sampling in Riyadh and grown to obtain the raw material. These included the following: *Ulocladium *spp., *Penicillium *spp., *Aspergillus fumigatus, Cladosporium *spp., and *Alternaria *spp. The raw material was processed under Good Manufacturing Practices for skin testing. Other commercially available outdoor (grass and tree pollens) and indoor (mites, cockroach, and cat dander) allergens were also tested.

**Results:**

The highest sensitization to indigenous pollens was detected to *C. murale *(32%) in Khartoum (Sudan) and *S. imbricata *(30%) and *P. juliflora *(24%) in the Riyadh region. The highest sensitization to molds was detected in Khartoum, especially to *Cladosporium *spp. (42%), *Aspergillus *(40%), and *Alternaria *spp. (38%). Sensitization to mites was also very prevalent in Khartoum (72%), as well as in Abu Dhabi (United Arab Emirates) (46%) and Jeddah (KSA) (30%).

**Conclusions:**

The allergenicity of several indigenous pollens and molds derived from autochthonous sources was demonstrated. Prevalence studies in different regions of KSA and neighbor countries indicate different sensitization rates to these and other outdoor and indoor allergens.

## 

Allergy is a global disease that is triggered, or influenced, by allergens present in the indoor and outdoor environments. Allergens vary from region to region, and some could be indigenous to a particular geographical location. The type, duration, and intensity of the pollination seasons of the different plants vary from country to country, depending on the climate and the vegetation. Although most studies have been conducted in European and North American countries, fewer studies have been performed elsewhere, especially using pollen extracts of local importance. Although pollen allergens have been characterized for the majority of the common grasses, weeds, and tree pollens, the allergenicity of pollen grains from other potentially allergenic plants has not been fully investigated. Aerobiological surveys are a fundamental tool for the control of pollinosis, and the proper knowledge of local aeropalynology is crucial to establish a correct etiological diagnosis.

Riyadh, the capital city of Saudi Arabia, is situated in the middle of the desert with very low relative humidity and high temperatures during the summertime (40°C to 50°C). However, artificial irrigation maintains grasses and weeds in some areas of the city. House dust mites (HDM) are found in negligible quantities. Jeddah is an ancient city located near the Arabian Sea. It has a very humid and hot (30°C to 40°C) weather. HDM are found in very high concentrations. Alkhobar and Dammam are new, connected coastal cities with high humidity and temperatures (40°C to 50°C) throughout the year. Al Ain city, United Arab Emirates (UAE), is known as a Garden city and has mild to moderate temperatures (30°C to 35°C) and abundant ornamental trees and plants. Khartoum, Sudan, is located in the central region of the country. It has a continental climate with low humidity and dry and hot (35°C to 45°C) weather conditions. Khamis Mushait is located in southwest Saudi Arabia. It has a mild climate and is surrounded by fields devoted to agriculture. It is a popular tourist destination in the summer due to its relatively mild summer weather and frequent rainfall.

Several aerobiology studies have been conducted in Saudi Arabia, indicating the potential allergenicity of locally abundant species [1]. These studies have confirmed that both local and imported florae were represented in air samples with Chenopodiaceae, grasses, and *Ambrosia *spp. as the most common botanical groups. The airborne concentration of airborne mold spores has also been investigated [2,3]. These studies concluded that *Alternaria *and several species of *Cladosporium *are of allergological importance in Saudi Arabia. *Cladosporium, Ustilago, Alternaria, Chaetomium*, and *Ulocladium *are the main mold spores detected in the outdoor environment in this region. *Cladosporium *emerged to be the most prevalent genus in the outdoor environment constituting up to 25% of all fungal spores in the dry region and 37.1% and 41.2% in 2 coastal cities, respectively. Among the species, *Cladosporium sphaerospermum, C. macrocarpum, C. cladosporioides*, and *C. herbarum *were the most relevant. Distinct seasonal fluctuations in mold spores were also detected [4]. The in vitro allergenicity of several of these species was also investigated, confirming the presence of the most important allergens [5].

Allergen sensitivity has also been investigated in the area using mostly commercially available batteries of skin tests and, in some cases, locally collected species [6-8]. The study by Almogren revealed that 75% of the patients reacted to 1 or more allergen extracts. The most frequently reacting indoor allergen was HDM (77.8%), followed by cat (33.6%) and cockroach (19.2%). Among the outdoor allergens, *Prosopis juliflora *was positive in 72.1%, Bermuda grass in 53.8%, *Chenopodium album *in 47.1%, rye grass in 36.5%, and *Salsola kali *in 36.5%. A significant proportion of patients (18.2%) also reacted to mold extracts. In a study in Kuwait, a total of 76.7% of the allergic patients had a positive reaction to *Salsola *pollen, while *Chenopodium album *was positive in 57.6% and Bermuda grass in 38.2% of the allergic patients.9 These studies have demonstrated the importance of the local flora in sensitizing individuals and inducing clinical symptoms.

Dust mites are the main indoor allergens in subtropical and tropical regions, and in general, in humid and warm climates. Sensitization to their allergens has been repeatedly associated with severe and persistent asthma [10]. In Saudi Arabia, there is considerable variation in temperature and humidity because of the influence of subtropical high-pressure systems and the many fluctuations in elevation. The 2 main extremes in climate are felt between the coastal lands and the interior. Along the coastal regions of the Red Sea and the Persian Gulf, the desert temperature is moderated by the proximity of these large bodies of water. Temperatures seldom increase above 38°C, but the relative humidity is usually more than 85% and frequently 100% for extended periods. This combination produces a hot mist during the day and a warm fog at night. These conditions are conducive for the proliferation of HDM. Several studies conducted in Saudi Arabia and neighboring countries have shown that HDM are important indoor allergens in this region [11-14]. However, one study that analyzed a small number of samples failed to identify HDM [15]. Cockroaches are also important sources of indoor allergens. One study has also suggested the potential allergenic role of cockroaches in Saudi Arabia [16]. In general, the better-studied species are *Blattella germanica *and *Periplaneta americana*, although *Blatta orientalis *is also an important source of allergens.

The diagnosis and treatment of respiratory allergic diseases is conditioned by the availability of representative allergen extracts containing the relevant allergens. The purpose of this study was to evaluate the prevalence of positive skin prick tests (SPTs) to pollen allergen extracts prepared with indigenous raw materials, as well as common indoor aeroallergens in patients having symptoms of inhalant allergies particularly allergic asthma and allergic rhinitis. A panel of 30 different allergens was selected on the basis of aerobiological studies conducted over years at 10 locations in Saudi Arabia.

## Materials and methods

### Patient Population and Participating Centers

A total of 492 consecutive patients having clinical symptoms of allergic respiratory diseases participated in the study. These patients resided in 6 different geographical areas, including the Kingdom of Saudi Arabia (KSA), the UAE, and Sudan. The number of patients entered per study location is shown in Table 1.

**Table 1:** Study Sites by City, Country, and Number of Participants

City (Country)	No. patients
Riyadh (KSA)	108
Damman (KSA)	103
Jeddah (KSA)	129
Khamis Mushait (KSA)	40
Khartoum (Sudan)	50
Abu Dhabi (UAE)	62

The following clinical centers participated in the study: (1) College of Medicine, King Saud University, Riyadh, KSA; (2) King Abdulaziz University Hospital, Jeddah, KSA; (3) King Fahad Medical Research Centre, King Abdulaziz University, Jeddah, KSA; (4) Elaj Medical Services, Riyadh and Khamis Mushait, KSA; (5) Children's Hospital, Riyadh Medical Complex, Riyadh, KSA; (6) King Abdulaziz Hospital and Oncology Center, Jeddah, KSA; (7) Maternity & Children's Hospital, Dammam, KSA; (8) Saad Specialist Hospital, Dammam, KSA; (9) the National Ribat University, Faculty of Medicine, Khartoum, Sudan; (10) Faculty of Medicine and Health Sciences, UAE University, Abu Dhabi, UAE; and (11) Al Imam Abdulrahman Bin Faisal Hospital, Dammam, KSA. This study was approved by the internal review board (Research Advisory Council under approval; RAC 2060 006).

The inclusion criteria were as follows: (1) seasonal rhinitis and/or asthma of at least 2-year duration based on the Allergic Rhinitis and its Impact in Asthma (ARIA)[17] and Global Initiative for Asthma (GINA)[18] criteria; (2) minimum of 5 years of residence in the study area; and (3) age between 14 and 49 years. The exclusion criteria were (1) nasal polyposis and (2) chronic obstructive pulmonary disease, autoimmune diseases, and malignancies.

A large percentage (> 80%) of the patients tested in the study were born in their respective areas and grew up close to the testing center/city. Other than in Riyadh, very limited numbers of immigrants from India or Pakistan are present in the population.

### Collection of Pollen From Autochthonous Plants

The selection of indigenous allergens was based on research findings from Riyadh and adjoining areas [1,6-8]. Indigenous raw material for pollen extracts was collected by Allergotek (Aerobiological Unit, King Faisal Specialist Hospital & Research Center, Riyadh) from the desert near the capital city of Riyadh, Saudi Arabia. The pollen was separated from the anthers and other debris using mechanical sieving. Indigenous plants from which pollen was collected included the following: *Chenopodium murale, Salsola imbricata, Rumex vesicarius, Ricinus communis, Artiplex nummularia, Amaranthus viridis, Plantago boissieri*, and *P. juliflora*.

### Collection of Spores From Autochthonous Molds

The selection of indigenous molds was based on previous research [2-5]. Mold spores were collected by Allergotek from cultures of the isolated molds grown in the laboratory. Spores for cultures were obtained from air sampling in Riyadh (KSA), as described [5]. The molds included in this study were *Ulocladium *spp., *Penicillium *spp., *Aspergillus fumigatus, Cladosporium *spp., and *Alternaria *spp.

### Manufacturing of Allergenic Extracts for Skin Prick Testing

The above collected pollens and mold cultures were dispatched to Inmunotek SL (Madrid, Spain). The rest of the allergenic starting material was supplied to Inmunotek by several international suppliers. The company was responsible for making the allergen extracts following Good Manufacturing Practices and standard operating procedures. Briefly, raw material was defatted, extracted in phosphate buffered saline, dialyzed in 5 kDa diafiltration membranes (Pellicon Filter Systems, Madrid, Spain), clarified, sterile filtered, and freeze dried. Afterward, the freeze-dried material was reconstituted with 50% saline/glycerol solution in sterile conditions to the desired allergen potency. Standardized and nonstandardized extracts were manufactured. Allergen extracts were standardized according to the Nordic Guidelines and expressed in histamine equivalent units [19]. Nonstandardized extracts were expressed in weight per volume (wt/vol) and protein and major allergen content, when available (see Table 2). SPT droppers (3 mL) contained indoor and outdoor allergens and positive and negative controls. Once properly packaged, allergen extracts were shipped to each participating center and stored at 4°C until use.

**Table 2:** Allergens and Concentrations Used

Chenopodium murale*	1:40 wt/vol, 100 μg/mL
*Cupressus arizonica*	50 HEP, 20 μg/mL
Salsola imbricata*	1:25 wt/vol, 100 μg/mL
Rumex vesicarius*	1:15 wt/vol, 60 μg/mL
Ricinus communis*	1:15 wt/vol, 70 μg/mL
*Olea europaea*	40 HEP, 70 μg/mL
*Morus alba*	1:80 wt/vol, 200 μg/mL
*Phoenix dactylifera*	1:35 wt/vol, 180 μg/mL
*Fraxinus excelsior*	1:60 wt/vol, 300 μg/mL
*Cynodon dactylon*	50 HEP, 500 μg/mL
*Phleum pratensis*	100 HEP, 400 μg/mL
*Lolium perenne*	100 HEP, 190 μg/mL
Artiplex nummularia*	1:20 wt/vol, 26 μg/mL
Amaranthus viridis*	1:70 wt/vol, 200 μg/mL
Artemisia monosperma*	1:40 wt/vol, 80 μg μg/mL
Plantago boissieri*	1:30 wt/vol, 170 μg/mL
Prosopis juliflora*	1:25 wt/vol, 200 μg/mL
*Dermatophagoides pteronyssinus*	50 HEP, Der p 1: 12 μg/mL
*Dermatophagoides farinae*	50 HEP, Der f 1: 7 μg/mL
*Blatta orientalis*	1:40 wt/vol, 800 μg/mL
*Periplaneta americana*	1:50 wt/vol, 1000 μg/mL
*Blatella germanica*	1:60 wt/vol, 1200 μg/mL
Cat dander	50 HEP, Fel d 1: 7 μg/mL
Camel dander	1:7 wt/vol, 150 μg/mL
Goat dander	1:5 wt/vol, 40 μg/mL
*Cladosporium* spp.	1:25 wt/vol, 11 μg/mL
*Aspergillus fumigatus*	1:30 wt/vol, 25 μg/mL; Asp f 1: 1 μg/mL
*Ulocladium atrum *	1:20 wt/vol, 15 μg/mL
*Alternaria alternata*	1:30 wt/vol, Alt a 1: 3 μg/mL
*Penicillium* spp.	1:30 wt/vol, 15 μg/mL

Mass units represent protein content.

*Pollens collected in the Riyadh region and considered indigenous.

HEP, histamine equivalent units.

### Skin Prick Tests

SPTs were conducted using disposable lancets on the forearms of the patients according to the guidelines of the European Academy of Allergy and Clinical Immunology [20]. Measurements were recorded after 15 minutes and scored as follows: mean diameter of the wheal < 3 mm = negative; ≥ 3 mm = mild positive (+); > 3 to 5 mm = moderate positive (++); > 5 mm = strong positive (+++); > 5 mm with pseudopods = very strong reaction (++++). Antihistamines were discontinued before the SPT was conducted. Glycerinated solutions of histamine phosphate (10 mg/mL) and saline were used as positive and negative controls, respectively. None of the patients was pretreated with drugs, which could affect the performance of the test.

## Results

### Indigenous Pollens

**Figure 1:**
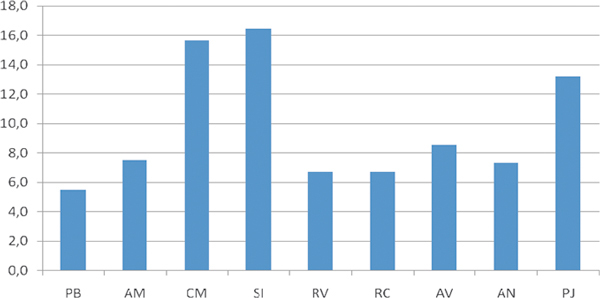
**Mean sensitization rates to the different indigenous allergens (N = 492)**. PB, *Plantago boissiere*; AM, *Artemisia monosperma*; CM, *Chenopodium murale*; SI, *Salsola imbricata*; RV, *Rumex vesicarius*; RC, *Ricinus communis*; AV, *Amaranthus viridis*; AN, *Atriplex nummularia*; PJ, *Prosopis juliflora*.

**Figure 2:**
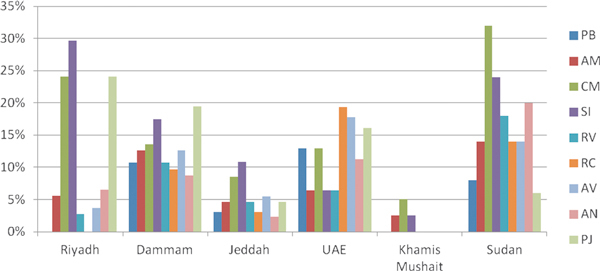
**Sensitization rates to different indigenous allergens in the different study sites**. PB, *Plantago boissiere*; AM, *Artemisia monosperma*; CM, *Chenopodium murale*; SI, *Salsola imbricate*; RV, *Rumex vesicarius*; RC, *Ricinus communis*; AV, *Amaranthus viridis*; AN, *Atriplex nummularia*; PJ, *Prosopis juliflora*.

Figure 1 shows the overall mean sensitization rate to the 9 indigenous allergens (ie, extracts from pollen collected in KSA) tested so far in the study population (N = 492). Overall, the highest mean prevalence of sensitivity to indigenous pollen allergens was detected to *S. imbricata *(15%); the highest sensitization rate to this allergen was 30% in Riyadh (KSA). Other highly prevalent allergens were *C. murale *(32% in Khartoum, Sudan) and *P. juliflora *24% in Riyadh (KSA) and 19% in Dammam (KSA). Figure 2 shows the mean sensitization rates to the different allergens in the different study regions.

### Other Pollens

**Figure 3:**
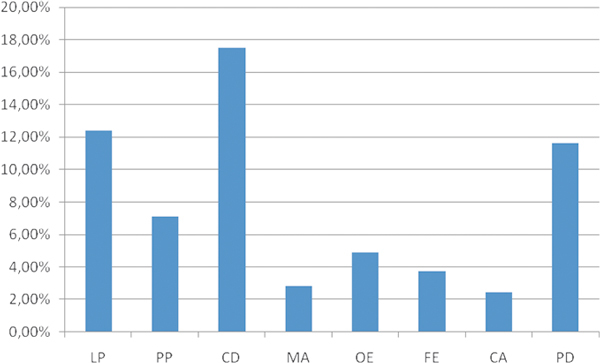
**Mean sensitization rates to different outdoor pollens, including grasses and trees, not considered indigenous (N = 492)**. LP, *Lolium perenne*; PP, *Phleum pretense*; CD, *Cynodon dactylon*; MA, *Morus alba*; OE, *Olea europaea*; FE, *Fraxinus excelsior*; CA, *Cuppresus arizona*; PD, *Phoenix dactyllifera*.

The sensitization rates to other pollens tested in this study are shown in Figure 3. As can be seen, there was considerable variation depending on the region. Three species of grasses were tested, but the results reflect sensitization the most prevalent one, which was *Cynodon dactylon *(mean overall sensitization rate of 19% across the region studied), ranging from 31% in Riyadh (KSA) to 3% in Jeddah (KSA). Among the trees, the most allergenic one was *Phoenix dactylifera*, reaching a mean total value of 12% and ranging from 35% in Riyadh (KSA) to 2% in Abu Dhabi (UAE). The mean overall sensitization rates for *Lolium perenne *and *Phleum pratense *were 11% and 6%, respectively.

### Indoor Allergens

**Figure 4:**
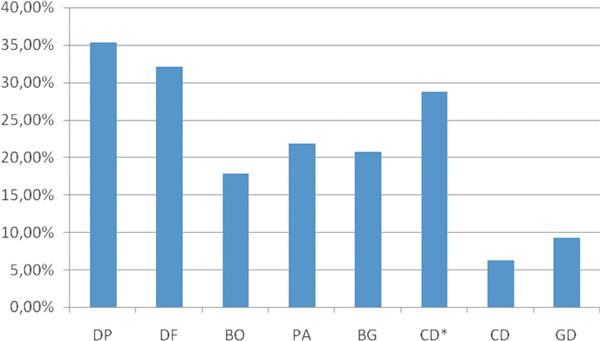
**Mean sensitization rates to the different indoor allergens (N = 492)**. DP, *Dermatophagoides pteronyssinus*; DF, *D. farinae*; BO, *Blatta orientalis*; PA, *Periplaneta americana*; BG, *Blatella germanica*; CD*, cat dander; CD, camel dander; GD, goat dander.

**Figure 5:**
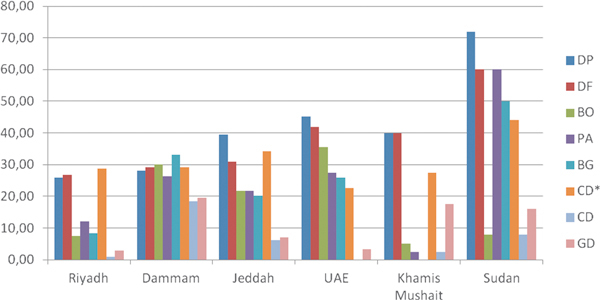
**Sensitization rates to indoor allergens in the different study sites (N = 492)**. DP, *Dermatophagoides pteronyssinus*; DF, *D. farinae*; BO, *Blatta orientalis*; PA, *Periplaneta americana*; BG, *Blatella germanica*; CD*, cat dander; CD, camel dander; GD, goat dander.

Figure 4 shows the mean percentage sensitization rates to indoor allergens. A more detailed description of the results is shown in Figure 5. Mites (*Dermatophagoides pteronyssinus *and *Dermatophagoides farinae*) were the main indoor allergens producing a positive skin test reaction, especially in Khartoum (Sudan), where sensitization to *D. pteronyssinus *and *D. farinae *were 72% and 60%, respectively, followed by Abu Dhabi, UAE (46%) and Jeddah, KSA (30%). Overall, cat was the third main allergen followed by the 3 cockroach species. In Khartoum, sensitization to *P. americana *was 60% and to *B. germanica *was 50%.

### Fungal Allergens

**Figure 6:**
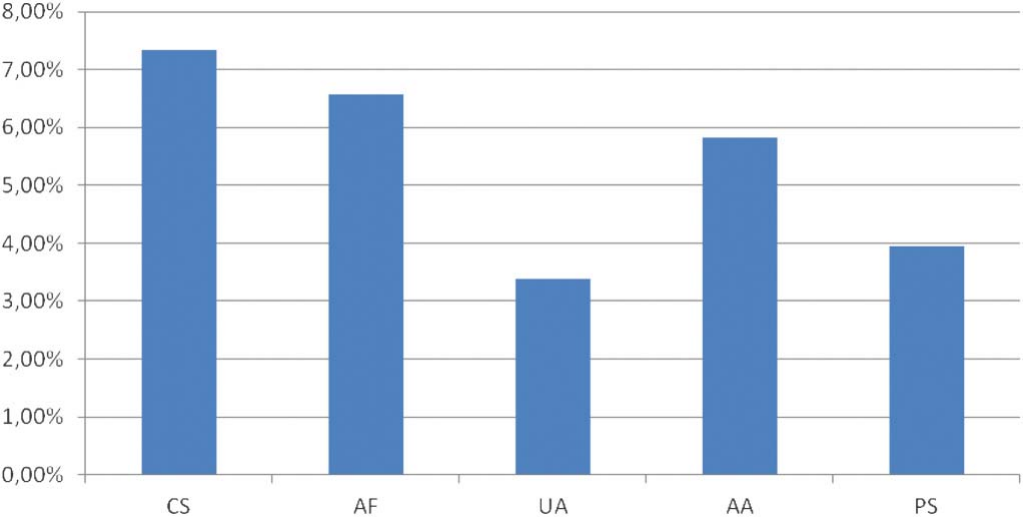
**Overall skin test sensitization rates to different mold species (N = 492)**. CS, *Cladosporium *spp.; AF, *Aspergillus fumigatus*; UA, *Ulocladium atrum*; AA, *Alternaria alternata*; PS, *Penicillium *spp.

**Figure 7:**
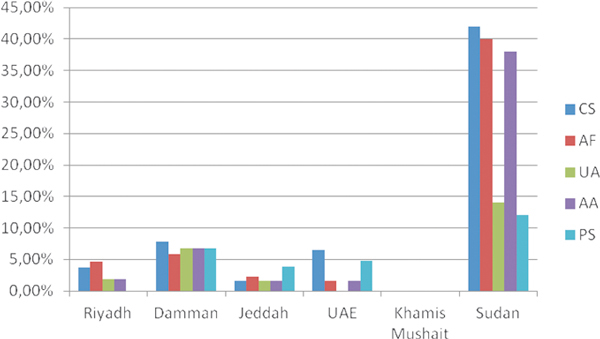
**Sensitization rates to mold species in the different study sites (N = 492)**. CS, *Cladosporium *spp.; AF, *Aspergillus fumigatus*; UA, *Ulocladium atrum*; AA, *Alternaria alternata*; PS, *Penicillium *spp.

Sensitization to fungal allergens is shown in Figure 6. As can be seen, there was a very different prevalence depending on the region. Sensitization to *Cladosporium *spp. varied from 0% in Abu Dhabi (UAE) and Khamis Mushait (KSA) to 42% in Khartoum (Sudan); sensitization to *Aspergillus fumigatus *from 0% in Khamis Mushait (KSA) to 40% in Khartoum (Sudan); sensitization to *Alternaria *from 0% in Khamis Mushait (KSA) to 38% in Khartoum (Sudan) (Figure 7). Other molds, *Ulocladium *and *Penicillium*, were less prevalent but also with big variations depending on the region studied (Figure 6).

## Discussion

This study describes allergen sensitization rates in 6 different regions of KSA, UAE, and Sudan using the same allergenic extracts. It is the first study of its kind to document SPT results using allergenic plants and mold species collected in the area. It provided a unique possibility of testing several indigenous plants, which had not been tested before. The study was extended to the Gulf region and neighboring Sudan to evaluate allergen sensitization to these allergens in Sudanese patients. It is interesting to note that the indigenous extracts reacted positively in a number of patients in the region. The extracts were tested in allergic patients by SPT. The test was conducted on a total of 492 patients evaluated at allergy units in different cities in the KSA, the UAE, and Sudan. Each participating physician and allergist conducted routine skin prick testing in their clinics and hospitals using the same diagnostic kits and the very same batch for each allergen. No side effects or adverse reactions were reported by any allergists/physicians during the study period.

The data collected in this study reveal valuable information regarding the diagnostic capacity of allergen extracts prepared with indigenous raw materials. Overall, patients were found to be comparatively more sensitive to indoor than to outdoor allergens. In this study, we have detected differences in the prevalence of sensitization to indoor allergens in Saudi Arabia, the UAE, and Sudan. The cultural habits and climate seem to have played a role. In Saudi Arabia and the UAE (with similar sensitization rates), socioeconomic conditions do not seem to influence the overall sensitization patterns because individuals in these 2 countries have the same habits (ie, rich and poor people in Saudi Arabia and the UAE use the same kind of dress, eat traditionally on the floor the typical Middle East food, and pray on the rugs). Some of these habits may not be true for Sudan. Therefore, it seems that some of the cultural habit may override the socioeconomic barriers. Furthermore, the group of patients who underwent skin prick testing in Khartoum belonged to the middle class/educated families.

Prevalence of sensitization to pollen allergens was variable in the region. Regarding grasses, 3 different species were tested (*C. dactylon, L. perenne*, and *P. pratense*). *C. dactylon *was the most frequent grass allergen in the area (18% overall), followed by *L. perenne *and *P. pretense *(12 and 8%, respectively). Sensitization to weeds was more important. Sensitization to *S. imbricata *was variable in the region, ranging from a quite high 30% in Riyadh or 24% in Khartoum to a lower 5% in Abu Dhabi or 2% in Khamis Mushait (KSA). These data support that *S. imbricata *is an important allergen in the area, as has been shown in another study performed in Kuwait [9]. In a study conducted in Oman, sensitization to *S. kali *was 34% in asthmatic patients,[21] which is similar to the sensitization rate of 30% detected in allergic patients in Riyadh (KSA). Whether sensitization to *S. kali *is due to cross-reactivity with *S. imbricata *should be further investigated because allergenic cross-reactivity between several *Salsola *spp. has been described [22]. Sensitization to other members of the Amaranthaceae and Chenopodiaceae families also seems prevalent in the region [23]. In this sense, sensitization to *C. murale *was 34% in Khartoum and 24% in Riyadh. Sensitization to tree pollen was less important than to weeds with the exception of *P. juliflora*. In this study, the highest rate of sensitization to *P. juliflora *was detected in Riyadh (24%) and Damman (20%). Other tree pollens, like palm tree (*P. dactylifera*) and olive tree (*Olea europaea*), seem to be less important (overall 12% and 5%, respectively), although they may reach local importance, especially in places where these species are more abundant. Other indigenous pollen extracts showing less prevalence (Figure 2) could be of clinical importance for the diagnosis of selected patients because strong reactivity was observed in certain patients.

Regarding molds, studies have repeatedly demonstrated that sensitization to fungi, such as *Alternaria alternata *is strongly associated with allergic rhinitis and asthma in children in the United States[24,25] and Saudi Arabia.2 Among asthmatic individuals in Kuwait, *Candida *spp. and *Aspergillus *spp. had the highest sensitization rates (23% and 21%, respectively), followed by *Helminthosporium *(19%), *Cladosporium *spp. (16%), *Alternaria *spp. (15%), and *Penicillium *spp. (14%) [26]. In Qatar, *Cladosporium *spp. (6 species, 40% of total fungi), *Alternaria *spp. (4 species, 21%), and *Ulocladium *spp. (4 species, 9%) were the main components of airborne fungi, and the commonest species were *C. sphaerospermum *(30%), *C. cladosporioides *(7%), *A. alternata *(14%), and *Ulocladium atrum *(5%) [27,28]. In general, *Cladosporium *spp. seems to be an important source of airborne fungal allergens in this region [3]. In the present study, sensitization to molds was also detected in some areas, especially in Sudan, where sensitization levels reached 42% to *Cladosporium *spp., 40% to *A. fumigatus*, and 38% to *A. alternata*. These figures can be considered as high.

Overall, the main indoor allergens were mites, being most predominant in the more humid areas. More than 70% of the patients analyzed in Khartoum were sensitized to mites. This figure is fairly high and consistent with sensitization levels obtained in allergic individuals in tropical and subtropical regions and in some more humid areas of Saudi Arabia, such as Jeddah [29]. A high sensitization rate was also detected to *D. farinae*, probably due to cross-reactivity with *D. pteronyssinus*. The prevalence of sensitization to mites in Riyadh was more according with sensitization rates to mites described in more dry areas [30,31]. Other important indoor allergens were the cockroach species (*P. americana, B. germanica*, and *B. orientalis*), which are also an important source of indoor allergens worldwide [32]. Sensitization to cockroach allergens has also been implicated as an important cause of asthma, especially in inner cities of the United States. Interestingly, in Khartoum, sensitization to *B. orientalis *was strikingly low in comparison to *P. americana *and *B. germanica *(Figure 5), despite the expected cross-reactivity within cockroach species [33,34]. Cat dander was the main animal allergen in the region (overall 28%), reaching to 43% in Khartoum. This value is similar to the one obtained in Jordania[35] and in other studies [36].

The present study shows a variable degree of allergen sensitization to various outdoor and indoor allergens in Saudi Arabia (Riyadh, Damman, Jeddah, and Khamis Mushait), the UAE (Abu Dhabi), and Sudan (Khartoum). These results suggest that indigenous allergens must be taken into account when considering the diagnosis, and even treatment, of respiratory allergies in Saudi Arabia and neighboring countries. The availability of indigenous allergen extracts for the allergists and patients is therefore greatly encouraged because they may benefit from a better and more relevant diagnostic tool and from a more specific immunotherapy in Saudi Arabia and adjoining Arab countries.

## Competing interests

The authors declare that they have no competing interests.
